# The Triadic Roots of Human Cognition: “Mind” Is the Ability to go Beyond Dyadic Associations

**DOI:** 10.3389/fpsyg.2018.01060

**Published:** 2018-07-09

**Authors:** Norman D. Cook

**Affiliations:** Department of Informatics, Kansai University, Osaka, Japan

**Keywords:** triadic cognition, polymodal associations, tool use, language, joint attention, harmony perception, pictorial depth perception

## Abstract

Empirical evidence is reviewed indicating that the extraordinary aspects of the human mind are due to our species’ ability to go beyond simple “dyadic associations” and to process the relations among three items of information simultaneously. Classic explanations of the “triadic” nature of human skills have been advocated by various scholars in the context of the evolution of human cognition. Here I summarize the core processes as found in (i) the syntax of language, (ii) tool-usage, and (iii) joint attention. I then review the triadic foundations of two perceptual phenomena of great importance in human aesthetics: (iv) harmony perception and (v) pictorial depth perception. In all five subfields of human psychology, most previous work has emphasized the recursive, hierarchical complexity of such “higher cognition,” but a strongly reductionist approach indicates that the core mechanisms are triadic. It is concluded that the cognitive skills traditionally considered to be “uniquely” human require three-way associational processing that most non-Primate animal species find difficult or impossible, but all members of *Homo sapiens* – regardless of small cultural differences – find easy and inherently intriguing.

## Introduction

The big psychological question in evolutionary theory remains as perplexing and as unanswered today as in Darwin’s lifetime: How can *Homo sapiens* be biologically so similar to other animal species and yet cognitively^1^ so different? In the 21st century, there has been a flood of books and articles on this topic. Notably, several concrete hypotheses have been formulated about the “mindful ape” concerning the emergence of (i) language, (ii) tool-usage, and (iii) social cooperation. These are the behaviors where human cognition appears to be most exceptional and consequently which have received the most consideration by many generations of scholars (e.g., Pasternak, 2007). Through a combination of conceptual insight and experimental ingenuity, significant progress has been made in specifying what is truly unusual about the cognition underlying those skills – and indeed which aspects are common to other animal species. Controversies are numerous, but one of the biggest obstacles in evaluating hypotheses concerning the human mind lies in the fact that human cognitive skills have blossomed into such complex behaviors that the “core” cognitive talents are far from obvious. In the reductionist tradition of the natural sciences, the search for origins has consequently focused on simplified phenomena – in animals, in infants, and most importantly in the reduced dimensions of laboratory cognitive science.

Two research strategies have become dominant. The first deals with differences in currently existing cognition among human adults, human infants, and various animal species, notably Primates. Interspecies comparisons in particular are notoriously difficult, but potentially provide a means to evaluate human behavior from a non-anthropocentric viewpoint. The second strategy is the study of the evolutionary record. As sparse and as inherently haphazard as the findings of paleoanthropology may be, fossils have the extreme merit of providing an unambiguous chronological sequence of the major events in the evolutionary history of our species (see **Appendix**: The Timeline of Human Evolution).

Both the experimental and the historical approaches have proven to be invaluable, but, whatever insights can be obtained, most researchers expect that the explanation of human cognition will be consistent with the known processes of biological evolution. In that respect, it is of interest that there is agreement among three of the most incisive modern thinkers on the cognitive evolution of *H. sapiens* regarding the step from pre-modern to modern mentality. That is, Donald (2001), Corballis (2011), and Tomasello (2014) have separately noted that, in accord with conventional evolutionary theory, the Primate brain could have undergone at most only *one* major “rewiring” in the transition from ape to human cognition over the relatively brief timespan that separates us from our pre-modern ancestors.

That revolutionary re-wiring may have been driven by innovative tool construction some two million years ago, the invention of language during an Ice Age survival crisis, or perhaps the emergence of social cooperation on the African savannah as our ancestors needed each other’s help to hunt together. Alternatively, the evolution of human “mindfulness” might have its origins in a more complex type of associational process that was then exploited in the development of our various cognitive talents. Several plausible hypotheses of this kind have been forwarded – often with a focus on tool-making and tool-using skills (Klein and Edgar, 2002; Corballis, 2011; Stringer, 2012; Tattersall, 2012; Suddendorf, 2013), sometimes with a focus on language (Bickerton, 1990; Jackendoff, 2002; Berwick and Chomsky, 2016) or speech (Jaynes, 1976; Lieberman, 2007), and sometimes with an emphasis on social cooperation (Deacon, 1997; Tomasello, 1999, 2014; Whiten, 1999; Saxe et al., 2004; Wrangham, 2009; Dunbar, 2016).

Not surprisingly, linguists have emphasized the supreme importance of language in the emergence of all types of characteristically human behavior. Without at least rudimentary language, they ask, what kinds of tool creation and group behaviors can realistically be expected to have occurred among our ape-like ancestors? In contrast, developmental psychologists and experts on animal behavior tend to see the inherently cooperative, social behavior of *H. sapiens* as the hallmark of our species. If, in times of crisis, our early ancestors came to empathize with one another and were inclined to find collective solutions to collective problems, then cooperative behavior may have preceded and motivated the subsequent development of tools and language. And, while acknowledging the importance of both language and social cooperation, paleoanthropologists understandably emphasize the long history of tool-making and tool-usage – and the unambiguous chronology of material artifacts. Specifically, the historical record on tools extends back 2 ∼ 3 million years, whereas tangible evidence of cooperative social activity and language is tenuous for all phenomena dating from more than 100,000 years ago.

Most scholars on human evolution would of course argue for the synergistic development of all three of these (and perhaps other) fundamental human skills (e.g., Deacon, 1997; Tattersall, 1998) – each contributing to the advancement of the others. But the sequence of evolutionary events and the precise nature of the “rewiring” of the human brain remain entirely speculative (cortical expansion? the addition of cross-modal sensory processing? the emergence of hemispheric specialization? the development of neuronal circuitry to sustain Boolean logic? etc.). Whether used first in tool-making, language, or social organization, once a new talent had become established, the novel capabilities of the newly wired human brain could then have been applied diversely to various modalities to enlarge the cognitive toolkit (Mithen, 1996, 2005) of *H. sapiens*. The alternative hypothesis to the “once-only revolutionary rewiring” of the human brain is the rather unparsimonious possibility of successive mutations that separately facilitated language, tool use, social cooperation, symbolic thought, face recognition, throwing, cooking, dance, music, art, and so on – with no real linkage among these human talents.

Efforts have in fact been made to enumerate the “universals” of human cognition (e.g., Brown, 1991), but Tattersall (2007), p. 134 has noted that

“the problem with such lists is that they can never be complete; there’s always something else to add… And none of these features in itself specifies anything about the human condition; we simply can’t know which of them, if any, is the ‘key’ human attribute, the one that was targeted by past natural selection.”

In the essay that follows, I summarize the case for thinking that five of the “universals” of human cognition that others have previously identified, emphasized, and described explicitly as “triadic” do indeed have a cognitive triad at their core. No attempt is made to delimit our triadic talents to these five phenomena alone, but they are, by consensus, arguably the most distinct and, moreover, the talents that researchers interested in animal cognition have the most difficulty relating to the full-blown talents of *H. sapiens*.

In the context of the types of essays published in *Frontiers in Psychology*, the present essay is clearly an “Opinion” piece – in attempting to bring together five highly contentious subfields of human psychology within a novel triadic hypothesis. At the same time, however, it can be said that the evidence indicating the importance of cognitive triads has already been presented by others in explication of the unusualness of human cognition separately in each of these subfields. In that respect, the present essay can be seen as a “Review” of current ideas in human cognition – with, to be sure, an emphasis on the supporting views of others that have focused on the perceptual/cognitive triads in language, tool-making, social cooperation, art and music. While I am unaware of any academic work that has argued explicitly against the triadic hypothesis, the vast majority of theorizing on the evolution of human cognition does not focus on “triads” – and, in that regard, the present work represents a personal “Opinion” that may or may not withstand the test of time. In any case, it may inspire further debate on the topic of “What Makes Us Human.”

Here, I outline the view that the “once-only” revolution in human cognition was the emergence of triadic neuronal processing – or the ability to handle the relationships among three items of information at the same time (Cook, 2012), as distinct from dyadic associations, i.e., simple binary correlations. By definition, triadic cognition includes both trimodal processing (where, for example, visual, somatosensory, and auditory information is used for task performance) and unimodal processing (where, for example, several distinct types of visual cue – occlusion, shadows, and perspective lines – each provide information for the understanding of visual depth). Stated as such, “triadic processing” is rather vague and in need of concrete explication. Fortunately, polymodal (multisensory, cross-modal) sensory processing has become a robust field of empirical research (e.g., Calvert et al., 2004; Murray and Wallace, 2012; Plaisier and Kappers, 2016), and the relationships among relevant cues in simplified perceptual tasks can often be specified in laboratory experiments and conclusions drawn concerning the relevance of dyadic versus triadic processing.

It is crucial for a proper understanding of triadic cognition to distinguish between the simple numerosity of perceptual/cognitive cues, on the one hand, and the complexity of the relationships among those cues, on the other. In earlier versions of the triadic hypothesis (e.g., Cook, 2012), I did not attempt a general definition of “threeness” under the assumption that the definition was self-evident. Prompted by reviewer comments, however, I now conclude that the “triad” in triadic cognition can and must be defined as the three *relationships* that are inherent to any set of three items. The numerosity of the cues themselves is not the issue, but research on short-term memory (e.g., Jonides et al., 2008), “chunking” (e.g., Cowan, 2001), and their development over the first few years of life (e.g., Oakes and Bauer, 2007) clearly indicates the involvement of both numerosity and causal relationships among items in memory in cognitive development.

That having been said, an inevitable confusion in the discussion of cognitive operations that involve small numbers of items, however, is the fact that – unlike all other sets – there are precisely three relationships among three items, whereas there is but one relationship between two items, already 6 relationships among four items, 15 among five items, and so on. In other words, no problems arise by conflating “items” and “relationships” in the case of three, but important differences do arise with any numerosity other than three. For the discussion that follows, the most convenient labels are those that indicate the numerosity of cues (dyadic vs. triadic, etc.), but the cognitive complexity arises from the number of distinguishable *relationships* among the cues.

In our own work in empirical musicology (Cook, 2002, 2009, 2017; Cook and Fujisawa, 2006; Cook et al., 2006; Cook and Hayashi, 2008; Fujisawa and Cook, 2011), and visual aesthetics (Cook et al., 2002, 2008a,b; Hayashi et al., 2007; Cook, 2012), we have manipulated the simplest of auditory and visual stimuli, and found that there is a dramatic leap in perceived complexity as one moves specifically from two to three sensory cues. In contrast, there is a trend toward increased complexity in the transition from three to four cues, or from four to five cues (etc.), but it is statistically rarely significant. In a word, there is something special in the auditory or visual “depth” of harmonies or images containing *three* (well-placed) tones or objects in comparison with only *two*. Recursively building on the perceptual triad by adding further auditory or visual cues is endlessly enriching (intriguing and aesthetically pleasing), but the leap from “sensation to art” appears to begin at the transition from the perception of 1 isolated correlation (inherent to 2 cues) to the perception of the 3 relationships (among 3 cues).

Having found empirical indications of the importance of specifically triadic processes in our own data, we returned to the literature (initially, on stimulus numerosity) in other fields where human “uniqueness” has been a traditional (if somewhat dubious) claim. In tracking the major evolutionary events that led from the mentality of our chimpanzee-like ancestors some 7 million years ago to the human mind today, it became apparent that others have stumbled onto similar cognitive “leaps” – sometimes using the labels of “triadic” vs. “dyadic” associations, but, more commonly, simply noting the inherent explosion of “complexity” as sensory cues proliferate. Hypotheses concerning the number of perceptual/cognitive processes that can be simultaneously held “in mind” are necessarily controversial^2^, but they are attractive in their conceptual simplicity and consequent empirical testability. In effect, the hypothesis of triadic cognition is both “radical” (in claiming to identify the cognitive functions underlying the transition from pre-modern to modern *H. sapiens*) and also surprisingly “conservative” (in being constrained by well-established findings in perceptual and cognitive psychology). While there remain several lacuna of unexplored issues, the basic hypothesis of triadic processing can be easily understood under the following five headings. There may indeed be other fundamental cognitive realms where human capabilities are qualitatively different (dance, cuisine, sports?), but the following are well documented in the literature on human evolution.

## Five Core Human Talents

Perhaps the strongest argument for the evolutionary origins of triadic cognition concerns the trimodal sensory processing required for manufacturing tools (Cook, 2012). That is, although the necessity of hand-eye (bimodal, i.e., proprioceptive and visual) coordination is often taken as the foundation of tool-*usage*, the auditory modality is known to provide precise *temporal* information (e.g., Coolidge and Wynn, 2009, p. 94) that is absent or less salient in other modalities – and that is particularly useful in tool-*making*. Because the manufacture of stone tools is by far the earliest concrete indication of modern human cognition, it is likely that the emergence of trimodal processing played a role in the emergence of triadic cognitive capabilities, in general. Specifically, as the brain began its expansion from 400 to 1400 cc (see the **Appendix**), unprecedented regions of cross-modal associations at the neocortical level emerged. In the modern human brain, the largest region of trimodal cortex can be found in the inferior temporo-parietal cortex (Glasser et al., 2016) (corresponding to a small region in the superior temporal sulcus of the chimpanzee brain), which partially overlaps with Wernicke’s area in the left hemisphere and a comparable region of trimodal convergence in the right hemisphere. These areas have been shown to be active bilaterally in a wide variety of cognitive tasks and their importance has often been invoked by theorists to account for “higher” cognition. Most notably, activity in these bilateral regions of trimodal association cortex are known to be the neocortical correlates of the Theory of Mind (Gweon and Saxe, 2013). While precise cortical localization of cognitive skills remains technically challenging, progress has been made in identifying the fundamental cognitive components that underlie human talents in language, tool-use and social interactions:

(1) Linguists since the onset of cognitive science in the mid-20th century have maintained that the use of language requires an understanding of syntax – most importantly, the sequential ordering of words to produce meaningful sentences. As first argued by Chomsky (1965), the ability to undertake syntactic transformations necessitates the use of *phrases* (at a minimum, two spoken words joined together through an unspoken “head”). As such, every phrase, every sentence consisting of phrases, and every syntactic manipulation involving phrases is inherently *triadic*. Linguists will of course note that there is much more to language, in general, and syntax, in particular, but the phrase is the widely accepted starting point for discussions of the apparently unique human talent for understanding grammar. Notably, the modern version of Chomsky’s theory, the Minimalist Program, explicitly maintains that “the core structure assembled by Merge consists of two syntactic objects plus a label” (Berwick and Chomsky, 2016, p. 136). These inherently triadic “phrases” are then implemented recursively to build hierarchically complex sentences, but, without exception, starting at the remarkably easy-to-understand level of simple triads (see the “Language” section below).
(2) Tool-use requires an understanding of the *three-way* relationship among (i) a tool, (ii) an object to be affected by the tool, and (iii) a concrete material context within which such manipulations can be productive (Johnson-Frey, 2004). Contrary to “common sense” notions from just 50 years ago, we now know that such understanding is not unique to our species (sea otters, capuchin monkeys, and chimpanzees exhibit a similar triadic understanding in limited tool-usage contexts). Tool-users of course remain a small minority in the animal kingdom, but the vast majority of the commonly cited examples of tool-use in the animal world are in fact dyadic (extensions of a grasping hand without a specific material context), rather than triadic. Similar to the role of phrases in language, the cognitive triad inherent to primitive tool-use barely scratches the surface of the human obsession with creating and manipulating highly complex material artifacts, but the story of tools clearly begins there (see the “Tools” section below).
(3) Social cooperation requires the “joint attention” of two participants on a common task. Cognitively, this has been described as a “triadic interaction” (Tomasello, 2003) among (at least) two communicators and their focus of attention. The basic idea is simply that people need to understand each other’s thoughts in order to coordinate differing actions directed at a common goal (Baron-Cohen et al., 2013). While animal studies provide interesting contrasts with human development, it is axiomatic that social cooperation is an extreme rarity in the animal world – and this has been most rigorously examined in the framework of the mother–child interaction. In developmental studies, the non-verbal behavioral responses of infants to small numbers of visual, auditory or haptic cues can be measured relatively easily and conclusions drawn about cognitive mechanisms (Gweon and Saxe, 2013). Whether or not “cooperation” in the sense of understanding the cognition of others actually occurs among hunting wolves or chimpanzees remains controversial (Tomasello et al., 2005), but it is worth recalling that the triad of “you, me, and our common goal” is something that human beings take for granted in virtually all forms of social activity. Easily said, but – evolutionarily – not easily accomplished (see the “Social Cooperation” section below).

Over the last century, the above three themes have been central to many discussions of the cultural evolution of *H. sapiens*. Because tools would necessarily have brought people together for common purposes, their importance for cooperative behavior and ultimately for the survival of the species is clear, but the causal relations among tools, cooperation and language remain uncertain. Moreover, the roles of several other unusual skills may also have played a role in human socialization on an evolutionary timescale. Particularly in light of the cave paintings and fossilized relics of musical instruments from more than 30,000 years ago, paleoanthropologists have speculated that art and music may underlie the enculturation of our ancestors into truly human communities. For this reason, scholars interested in rather high-level aesthetic issues have been able to ask concrete questions concerning what cognitive capacities underlie both the “art instinct” (Turner, 2006; Dutton, 2009; Davies, 2012; Chatterjee, 2014), and the “music instinct” (Blacking, 1973; Storr, 1992; Addis, 1999; Ball, 2010). Specifically:

(4) An appreciation of all types of music that use auditory pitch requires an understanding of harmonic mode (Meyer, 1956) – either the familiar major and minor modes used worldwide in folk, popular and classical music or the somewhat less-familiar unresolved “tension” mode of atonal music, jazz and the trance-like music of the Javanese gamelan. We know from a century of empirical study in music psychology that the emotional tone of music is established primarily by harmonies – played either as melodies or as chords – and always consisting of a minimum of *three* distinct tones (see the “Harmony Perception” section below). [Note that the creation of rhythms – as distinct from an underlying “beat” – also requires a minimum of *three* pulses (Cooper and Meyer, 1960; Desain and Honing, 2003), but clarification of the puzzle of harmony has been the dominant theme in traditional music theory]. While paleontologists might argue that music has far less evolutionary significance than, for example, the practical crafts of tool-making, nonetheless, participation in group activities involving music would have provided unprecedented social cohesiveness as group members – constrained by the vocal harmonies and rhythms of music – learned how to cooperate with each other in non-survival musical pursuits.
(5) An understanding of most representational visual art requires a capability for so-called pictorial depth perception: the ability to perceive the *illusory* 3D structure of scenes depicted in 2D pictures (Gombrich, 1961; Arnheim, 1974; Kemp, 1990). Clearly, to see the “animals” painted on a cave wall requires that the viewer *not* attend to the irrelevancies of the actual material setting – but rather focus on a fundamentally “unrealistic” static representation of visual objects – despite an abundance of contradictory sensory cues. The viewing of pictorial art in the frighteningly reduced perceptual conditions of cave art (Curtis, 2006) was an unprecedented act of joint attention focusing on the visual modality. Although art theorists tend to view the aesthetic talent of pictorial depth perception as a “high end” modern skill, the perception of 3D structure in paintings is (with minimal exposure to such art) universal among *H. sapiens* and, conversely, a rarity among animal species. Some of the visual cues that signal spatial depth are essentially dyadic (relative size, relative height, occlusion of one object by another) – and are perceived by many species. But the techniques known as linear perspective and chiaroscuro (the artistic use of shadows and shading) are demonstrably triadic in relying on the presence of a minimum of three independent visual cues to give static 2D visual images an illusory depth dimension “into” the canvas (Hecht et al., 2003) (see the “Pictorial Depth Perception” section below). In perceiving those cues, the relative depth of objects depicted “in perspective” is understood by all normal human observers, but remains perceptually opaque to most animal species.

Clearly, the evolutionary and cognitive research strategies in art and music involve distinct sensory modalities and address phenomena that are very different from language, tools, and social cooperation, but the possibility of a common triadic interpretation of the underlying cognition is of deep theoretical interest. Visual art without an illusory depth dimension is of course possible [“flatism” in abstract art was in vogue in the early 20th century, (e.g., Wolfe, 1975)]. Furthermore, music that avoids or minimizes major and minor harmonies can be created with some effort (e.g., Schoenberg, 1978), and produces some intriguing tonal effects. Nevertheless, the vast majority of modern-day artists and composers devote most of their time and energy specifically to the enhancement of the “illusory” features made possible by manipulations of triadic cues. In both abstract and realistic visual art, the illusion of 3D structure on a 2D canvas and, in both high-brow and popular music, the ebb and flow of illusory affect utilizing major and minor harmonies are crucial to the evocation of aesthetic pleasures. While other dimensions in art and music are of interest, it remains as true today as 500 years ago that illusory spatial depth and illusory musical emotions are two of the most important and technically most difficult issues for artists and musicians to master.

The inevitable questions arise: In the long road of Primate evolution, were our ancestors of less than 100,000 years ago the first to understand tertiary relationships in the auditory and visual modalities? Does the cognition inherent to aesthetic perceptions in art and music reflect the cognitive leap into human “mindfulness” – exemplified by earlier developments in tool-making, social cooperation and linguistic syntax, but also realized in aesthetics? Is the human brain cognitively unusual principally in its fluent capabilities for polymodal associations? Definitive answers are not yet possible, but the hypothesis of triadic associations as the gateway to “higher cognition” can be easily summarized. Below, the processes at the heart of music and art are first examined, and then the traditional evolutionary questions concerning language, tools, and social cooperation are once again considered.

## The Core Triads

### Harmony Perception

Psychophysicists in the mid-19th century found that the pleasantness (“consonance”) of two-tone intervals was a consequence of not simply the difference in frequency between two fundamental pitches (say, middle C and the G above it), but also a consequence of the frequency differences among all combinations of their overtones (von Helmholtz, 1885/1954). It then became surprisingly easy to explain quantitatively (Plomp and Levelt, 1965; Sethares, 2005) why some intervals are consonant (and readily incorporated into music of all kinds), while other intervals are starkly dissonant and rarely a part of popular music. The psychology of such musical dyads is now well understood (Parncutt, 1989; Huron, 2006), and constitutes a foundational concept in the science of music. That being said, the perception of pitch intervals alone does not explain the puzzle of harmony. When three (or more) tones are played simultaneously, their overall “sonority” is quite clearly *not* computable from the sum of their interval consonances. Somehow, pitch triads – whether played as melodies or chords – introduce new musical properties that are absent in two-tone intervals. Nineteenth and 20th century psychoacoustics left this problem largely unaddressed, but in recent years the nature of harmony has been addressed explicitly in terms of three-tone psychoacoustics (Cook, 2009, 2017; Tymoczko, 2011).

**FIGURE 1:**
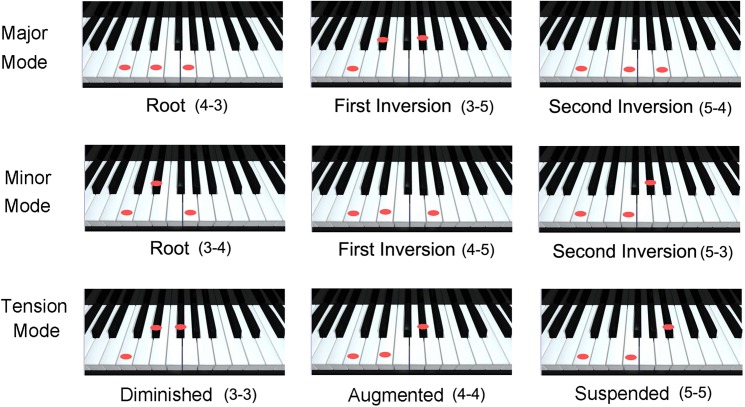
Examples of the interval substructure of the most familiar major, minor, and “atonal” tension triads. While three-tone combinations do not exhaust the possibilities of polyphonic music, these three modes are the minimal musical expression of positive, negative and unresolved, ambiguous affect through harmony. Note that all of the pitch intervals illustrated here are consonant, consisting of three, four or five semitones, but their relative positions lead to the very different affective implications that are associated with each mode.

Importantly, it is not until pitch *triads* are encountered that the characteristic emotional twinge of the major and minor modes is experienced. Harmonic triads are where music begins to become interesting because even simple harmonies are the affective triggers, used in nearly all types of music, that make music emotionally meaningful. If we include the “atonal mode” heavily employed in modern classical and jazz styles, then moment-by-moment musical compositions can be described in terms of their ability to elicit the positive or negative emotional responses of the major or minor keys or, alternatively, to deliver us into a state of unresolved tension through atonal melodies and harmonies. Note that most of analytic music theory is concerned with more complex notions of tension and release, expectation and resolution, and the ebb and flow of relatively large musical phrases (Narmour, 1990; Temperley, 2007), but the basics of harmony can be described already at the level of three-tone combinations (**Figure 1**).

Although the concept of musical “mode” was an important insight in the early Renaissance, traditional ideas in music theory failed to provide an explanation of *why* quite simple three-tone melodies or chords typically elicit emotional reactions. On the one hand, it was understood that the relatively “stable,” “sonorous” and “beautiful” (and, as a consequence, most frequently used) major and minor chords contain structurally *asymmetrical* three-tone combinations (intervals of three, four or five semitones), whereas the less stable tension chords contain an abundance of *symmetrical* triads (two neighboring intervals containing the same number of semitone steps) (**Figure 1**). The unresolved character inherent to *symmetrical* triads was already noted by Vincenzo Galilei (the astronomer’s father) in 1581 (Heilbron, 2010, p. 10), and was explained on the basis of Gestalt psychology nearly four centuries later by Meyer (1956), but quantitative acoustical models are a new development in the 21st century.

We have exploited the fundamental insights of Galilei and Meyer to develop algorithms for calculating not only the overall sonority of chords, but, more significantly, the major, minor or tension “valence” of any combination of three (or more) tones (e.g., Cook, 2002, 2009, 2012, 2017). Specifically, valence can be calculated on the basis of three-tone (two interval) pitch structures. Our theoretical innovation was based simply on the fact that, within the major chords, there is an abundance of three-tone structures where the lower interval is larger than the upper interval (e.g., 4&3 and 5&4 semitones), and vice versa for the minor chords. As noted by Galilei, the tension chords, in contrast, show an abundance of three-tone structures containing equivalent intervals (e.g., 3&3, 4&4, etc.). Note that such patterns are insufficient for calculating the harmonic mode if only the fundamentals (i.e., the notes actually played with one’s fingers on a musical instrument) are considered. By bringing the higher harmonics of the fundamentals into the algorithm, however, the quantitative results are unambiguous (Cook and Hayashi, 2008) in distinguishing among major, minor, and tension modes.

The radically simple conclusion drawn from the regularities of three-tone harmonies is that there is an *acoustical* basis for the worldwide popularity of the stable major and minor chords relative to the (interesting, provocative, but rather) unstable tension chords (diminished, augmented and suspended triads). In other words, the popularity of the major and minor triads need not be dismissed as ineffable, aesthetic phenomena of arbitrary cultural origin, because both their overall sonority and their positive/negative valence can be calculated directly from definable acoustical properties (Cook, 2017).

Although a cultural interpretation of “Western” harmony is currently fashionable, we have shown that the emotional “tug” of both melodies and harmonies is based on two acoustical principles. The first concerns the familiar notion of *dyadic* dissonance (where chords containing certain intervals are simply avoided). The second concerns the harmonic sonority and modality of *triads* (discovered by Italian Renaissance musicians, developed independently in the Raags of Northern India, and invented and reinvented countless times in various folk traditions). The atonal tension triads are typically used to enhance the unresolved ambiguity of pitch combinations, and then subsequently resolved to either the major or minor mode by pitch rises or falls.

**FIGURE 2:**
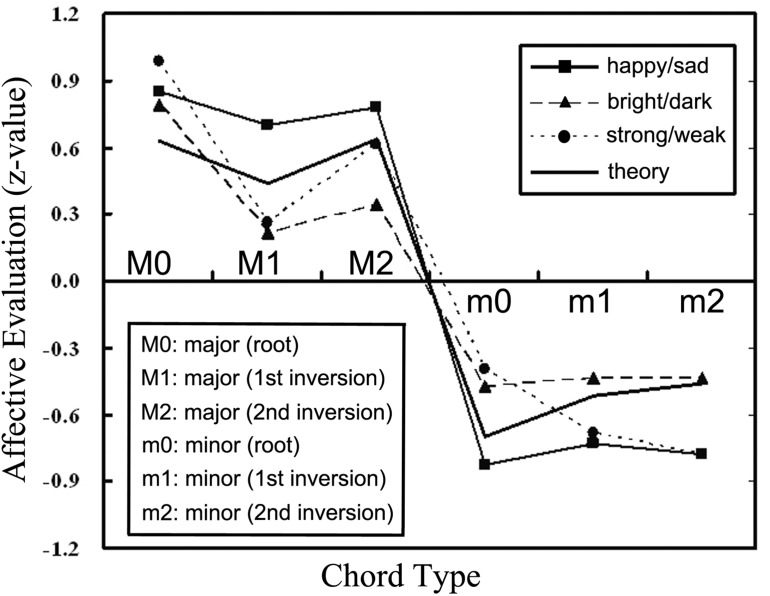
Evaluation by non-musician undergraduates of major and minor chords according to their perceived happy/sad, bright/dark, strong/weak character (Cook, 2012, p. 66).

It should be noted that the “mystery” of the positive/negative affect of simple harmonies is *not* explained solely by the avoidance of small dissonant intervals. Why are triads with interval structure 3&4, 4&5, or 5&3 semitones heard as minor, whereas 4&3, 3&5, or 5&4 are heard as major? The properties of isolated dyads provide no insight, but, as noted above, the size *ratio* of upper and lower intervals in pitch triads (among all fundamentals and partials) provides quantitative answers. As a consequence, it can be concluded that the classification of any chord as major, minor or tension has an acoustical basis (Cook, 2017). The human “ear” can perceive such ratios, as has been demonstrated experimentally both for musicians and non-musicians, for adults and children as young as 4 years, and for Easterners and Westerners – all of whom can reliably distinguish among major, minor, and tension harmonies (Roberts, 1986; Kastner and Crowder, 1990). Typical results from our laboratory for the major and minor chords are shown in **Figure 2**.

Let it be noted that “real music,” as distinct from the minimalist auditory stimuli used in psychoacoustical experiments, is incomparably more complex when contextual effects are considered, but the core harmonic phenomena are explicably triadic (determined by the ratio of two intervals), and no longer an aesthetic “mystery” left over from the Renaissance.

## Pictorial Depth Perception

In the visual arts, the techniques of linear perspective and chiaroscuro can provide a strong and coherent illusion of 3D structure in scenes depicted on a 2D canvas – an artistic effect that was generally absent prior to the 15th century. Interestingly, the near-universal human enthusiasm for believing that we actually “see” the 3D depth implied in the 2D picture is not shared with most other species – trained chimpanzees and gorillas being important exceptions. The implication is that, while capable of recognizing 2D shapes on a 2D surface, most animals do not interpret those shapes as representing objects in an imaginary (pictorial) 3D world. They are, of course, literally correct in *not* confusing the 2D picture with 3D reality, but, for that same reason, they fail to understand, enjoy, and utilize the fantasy world of 3D space represented on 2D surfaces that makes visual art and graphical design both interesting and useful for *H. sapiens*.

So, just what is the cognitive mechanism that underlies our “clever gullibility” to see 3D structure in 2D paintings? The answer, in a word, is perspective. Discovered and developed as an artistic technique in the early Renaissance, the theory of perspective is surprisingly complex, still debated by specialists (Kubovy, 1986; Damisch, 1993; Panofsky, 1997; Massey, 2003; Edgertown, 2009) and still the focus of much artistic invention. Although the hyper-geometric realism of 16th century European artists is no longer fashionable, the techniques for depicting realistic – or, at least, geometrically recognizable – solid, 3D objects on flat canvases is as alive in visual art as is the use of harmony in music.

**FIGURE 3:**
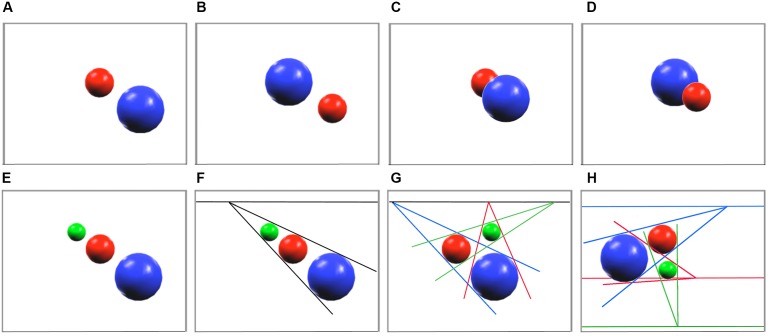
In experimental tests of pictorial depth perception, undergraduates are asked to indicate which ball appears nearest to them (Cook, 2012, pp. 133–147). It is found that the depth structure of a scene containing just *two* balls is inherently ambiguous **(A,B)**, unless a third component – a relationship of occlusion **(C,D)** is included. In contrast, the depth interpretation of *three* non-overlapping objects **(E–H)** is sensitive to their linear (mis)alignment. Random placement of the objects **(H)** leaves a multitude of inconclusive dyadic comparisons (relative size, relative height) that, more often than not, are mutually contradictory with regard to the implied vanishing point and implied (red/green/blue) horizon lines. Perspectival alignment solves that problem and implies a coherent depth interpretation with a unique horizon line and unique vanishing point **(F)** or a unique horizon line only **(G)**. In all three cases **(E–G)**, an illusion of depth is created (see text).

Moreover, we now know from experimental work on the elements of perspective drawing that the inference of an illusory third dimension is made possible by the alignment of quite small numbers of visual cues (Zeki, 1999; Solso, 2003; Cook, 2012). As shown in **Figure 3**, the depth relationship between two *non-overlapping* shapes in a 2D picture (A, B) is inherently ambiguous (and responses are typically slow and variable in evaluating the spatial configuration). But, if there is *overlap* (“occlusion”) of one shape on the other (C, D), the depth configuration becomes obvious to all normal observers. There are then two objects *plus* an explicit relationship between them. Regardless of the relative size or relative vertical position of the objects on the 2D canvas, the occluding shape is seen to be closer and the occluded shape is perceived as further away: an illusion of “depth” has been created.

Unlike those dyadic examples, the depth perception of (non-overlapping) triads of shapes is more subtle. Specifically, the relative positioning of all three shapes on the 2D canvas determines the perception of the depth structure of the scene (**Figures 3E–H**). There is a strong inference of depth when at least *three* shapes (as distinct from two) lie in the extremely low probability configuration of linear alignment on a 2D plane (F). If the difference in size of the three objects (taken two at a time) produces convergence of perspective lines on a common horizon line, then a depth interpretation is again favored. However, a random distribution of three similar shapes of different sizes would *not* imply a gradual decrease in size with distance and *not* imply a unique vanishing point on the horizon (H). Therefore, the “chance” *alignment* of three such shapes – with (F, G) or without (E) drawn perspective lines – is correctly inferred to be a highly significant (low probability) arrangement.

In contrast, *two* shapes of different size (A, B) can *never* be “unaligned” in pictorial depth: it is necessarily the case that perspective lines joining the edges of, for example, two spheres converge and create a vanishing point on the horizon. As a consequence, the fact of their convergence (on or off of the canvas) provides no information: the probability of convergence is 1.0 and the likelihood of that particular configuration in 3D space cannot be calculated. The convergence of the six lines that join three shapes drawn in linear perspective tells a very different story. Their meeting at a unique vanishing point (F) or on a unique horizon line (G) is such a low probability occurrence that the human brain normally infers that there is a cause (alignment in depth) for these linear relationships.

Such findings are consistent with the ideas of Purves and Lotto (2003) on the effects of *a priori* probabilities in visual perception. In brief, the depth perception of visual scenes containing *two* non-overlapping shapes (A, B) is inherently uncertain, but there are both low-probability (E–G) and high-probability (H) configurations of scenes containing *three* non-overlapping shapes. On the basis of accumulated visual experience, the human brain detects low-probability events – and (unlike most animal brains) automatically draws (depth) conclusions from the apparently non-random alignment of the visual cues on a 2D canvas.

**FIGURE 4:**
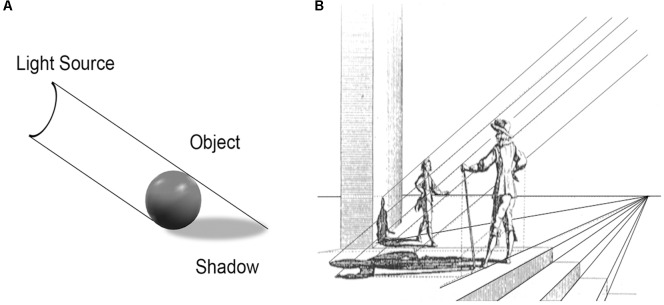
**(A)** The triadic nature of cast shadows can be seen in the alignment of the shadow with an opaque object and a light source. **(B)** As illustrated by many Renaissance artists, such as DuBreuil in 1654, such linear relationships underlie the realistic depictions of both shadows and perspective.

Also beginning in the Renaissance, artists have explored the effects of depicting objects with realistic shading and shadows. The utter simplicity of the perceptual triad underlying cast shadows is well known (**Figure 4A**), and has often been noted by commentators on the artistic use of shadows using examples from Renaissance art and astronomy (Baxandall, 1995; Gombrich, 1995; Stoichita, 1997; Casati, 2004). That is, if a cast shadow is visible, there *necessarily* exists an opaque object in line between the surface on which the shadow is cast and the light source. That *triadic* relationship provides a wealth of information on the structure of the visual scene for a brain engaged in calculating the probabilities of depth relations (Purves and Lotto, 2003) on the basis of the static picture. Unlike the visual systems of most animal species – that rely exclusively on binocular stereopsis and monocular motion parallax to estimate depth, the human mind has learned to interpret the so-called “monocular” pictorial depth cues to decipher the 3D structure in 2D pictures.

Already in the Renaissance, artists fully understood that the laws of linear perspective needed to be applied consistently over the entire canvas in order to create a convincing illusion of 3D structure. Starting with the two triadic principles of (i) parallel lines to depict shadows (**Figures 4A,B**) and (ii) converging lines to convey distance to an illusory vanishing point (**Figures 3F,G**, **4B**), their repetitive use produced unprecedented (Edgertown, 2009) spatial realism in 2D paintings. Subsequent generations of artists have come to emphasize other visual qualities, such as color and texture, but the vast majority of fine art displayed in museums worldwide has been created explicitly to convey some degree of illusory 3D structure on 2D surfaces using the “tricks” of linear perspective and chiaroscuro.

Although the Renaissance formalization of the artistic techniques for producing illusory depth illusions was a huge intellectual insight, the perceptual capacity came much earlier to *H. sapiens*. Judging on the basis of the pre-historic cave paintings in France and Spain (Curtis, 2006), at least some of the principles of pictorial depth perception were already understood by our ancestors more than 20,000 years ago, but are still unshared with most animal species. Similarly, the positive and negative affect of the major and minor modes was undoubtedly perceived many millennia before the Renaissance invention of simultaneous chords, but empirically we know that animals – including chimpanzees and songbirds – cannot be successfully taught the “happy–sad” illusion of harmonic mode (e.g., Hoeschele et al., 2012).

Having reached some tentative conclusions concerning the effects of triads of cues in the high-level perception of both music and art, we have asked the obvious next question: Are other of the “unique” talents of our species also a consequence of triadic cognitive processes? The answer is necessarily complex and will eventually require support from brain-imaging studies for general acceptance, but there is already considerable evidence in the psychological literature suggesting the importance of triads in specifically human cognition. Below, the triadic insights that have already been pointed out in empirical studies of language, tool use, and social cooperation are reviewed.

## Language

**FIGURE 5:**
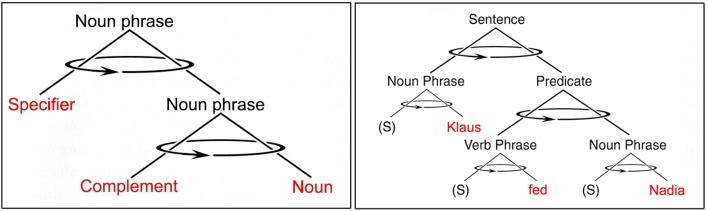
Phrases are cognitive triads consisting of pairs of spoken words (in red) joined through an unspoken “head.” On the left is shown the structure of a noun phrase that includes a specifier, a complement, and a noun (e.g., “a nice tune”). On the right is shown the recursive phrase structure of an entire sentence (e.g., “Klaus fed Nadia”) with optional specifiers “(S)” omitted. Arrows indicate possible phrase rotations.

The cognitive triad that lies at the heart of modern linguistic theory is the “phrase” – advocated since the 1950s by Noam Chomsky in the form of “transformational grammar” (1965) [later called “head-driven phrase structure grammar” (Pollard and Sag, 1994) in recognition of the central role of head-rotation]. Note that the latest incarnation of transformational grammar is now labeled the “minimalist program” (Boeckx, 2006), and is an attempt to reduce triadic phrase *structures* to multiple dyadic “merge” *functions*. I agree with both Bickerton (2014) and Tomasello (2014) that the emphasis on dyadic “merging” is a possible alternative expression of phrase structure, but is perhaps an unnecessary confusion that detracts from more than 50 years of linguistic theory based on phrase structure. Although coherent explanations of linguistic principles can follow from either the dyadic merge mechanism or the triadic phrase structure, the traditional emphasis on phrase structure greatly facilitates an explanation of the generality of triadic mechanisms in the “higher” cognition of *H. sapiens*. In either case, a coherent theory of syntax has already been built upon the linguistic insight that every phrase (noun phrase, verb phrase, prepositional phrase, etc.) entails the “merging” of two words through a connecting “head” (**Figure 5**).

The task that all language users repeatedly face when producing or hearing speech is to determine the unique meaning that corresponds to a specific combination of words organized into such discrete phrases. English-speakers pay attention primarily to the word-order within and between phrases. In other languages, the prefixes and suffixes of words and their agreement among the parts of speech can be more important than word-order, but in all languages comparable rules of syntax must be followed to indicate the relationships among words organized into phrases with specific – normally unique – meanings. As Bickerton (1990, p. 59), has noted, human beings “have a kind of template or model of what a phrase must be like. Not just a noun phrase: any kind of phrase. For the remarkable thing is that phrases of all kinds… are constructed in the same way. A phrase consists of three parts.” What Bickerton calls the phrase “template” is the foundational cognitive triad on which all of language is built. Without triadic structures, we (and all animal species) have only an amorphous soup of associations with no possibility of coding or decoding precise causality. Understanding the meaning of two nouns and one verb (e.g., **Figure 5**, right), we immediately know of the kinds of events that might be conveyed through such language, but without a familiarity with the arbitrary rules of phrase-ordering, we do not know who did what to whom. Dyadic associations do not suffice for explicating causality.

**FIGURE 6:**
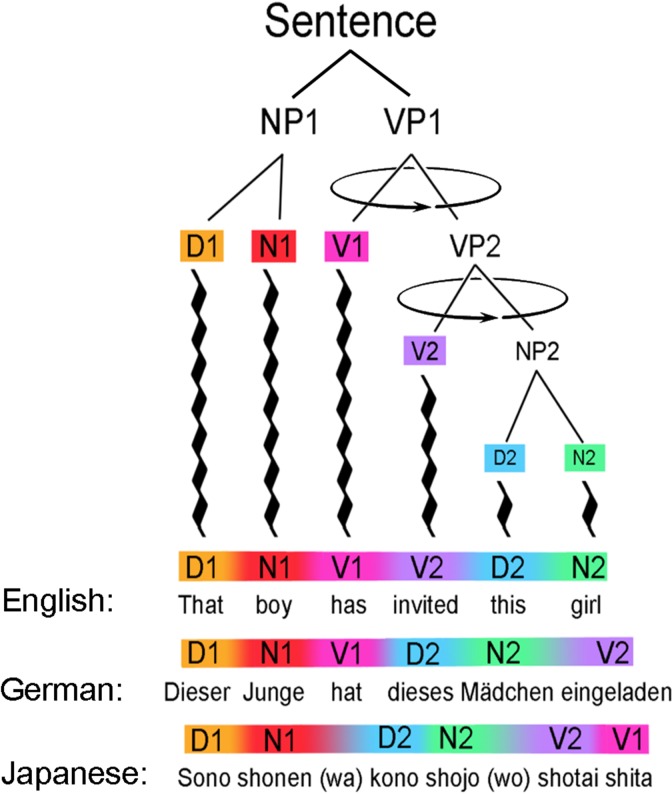
Depending on the arbitrary rules of different language communities, the same meaning can be translated into a foreign tongue by rotating phrases (NP1, NP2, VP1, VP2, etc.) around their heads, like a mobile twisting freely in space. Serial lexical replacements will normally *not* suffice for translation, but lexical replacements *plus* phrase rotations will often succeed. Here, an English sentence can be transformed into German by rotation of the VP2 phrase, and the German into Japanese by further rotation of the VP1 phrase.

In triadic phrases, words are necessarily connected two at a time in a temporal sequence (because of the linear ordering demanded by speech), but the human ability to understand the “chunking” of lexical units into phrases is still a deep mystery. According to Chomsky (2000), language capabilities are hardwired – and as “instinctual” as seeing the depth in a flat picture or hearing the emotional ring of a simple melody. Interestingly, the assignment of the order of the spoken words in each phrase is clearly *not* hard-wired, but learned – language-by-language, individual-by-individual, sentence-by-sentence (Evans, 2014). As most people know from the experience of studying foreign languages, the sequence of words in phrases is as arbitrary as the momentary linear order seen, for example, in a Calder mobile (**Figure 6**).

In other words, while the ability for phrasal “chunking” may be inborn, syntax is certainly *not* instinctual at the level of word-order. Indeed, in the world’s ∼6000 languages, every possible sequencing of subject (S), verb (V) and object (O) is used as the default structure. Most (90%) begin with subjects (SOV and SVO), but verb-initial languages (VSO and VOS) are not uncommon (Hawaiian and Celtic languages) and sentences beginning by default with direct objects are also known (Carnie and Guilfoyle, 2000). For any given language, there are often uniquely correct sequences, but the “correct” sequence is generally different in, for example, German, English, and Japanese – and translated into one another by means of phrase rotation. What remains constant across all languages is the presence of phrasal units that can be arranged recursively into larger-scale phrases and ultimately whole sentences.

With locally agreed-upon rules of sequencing, individual phrases have “correct” or “incorrect” temporal order to convey a specific meaning, but they can be rotated at will to agree with the sequencing rules of other languages to produce, once again, meaningful sentences with unambiguous semantics. Moving an adjective from its position before a noun (as in English) to after it (as in Thai), or transplanting a verb from its early position in English to its end position in Japanese or Latin may seem “unnatural” to English speakers, but those are precisely the kinds of syntactic rules that every young child absorbs from a language community, and soon masters. Because of such syntactic variability, successful translation therefore requires more than a one-to-one replacement of words with their lexical equivalents in a foreign tongue. The more challenging syntactic task (for second language learners) is to rotate the branches in a linguistic tree so that the same meaning is conveyed in a different language – often using a radically different sequence of spoken words (**Figure 6**).

Where do other species stand in their understanding of language? Remarkably, chimpanzees can learn the meaning of several hundred arbitrary symbols (Savage-Rumbaugh et al., 2001) and minor birds are astoundingly capable phoneticians (Pepperberg, 1999). But can these species learn syntax and, specifically, do they detect the semantic significance of phrase structure? The academic debate is far from resolved, but there is one issue concerning which the empirical data are clear. Analysis of the “utterances” of chimpanzees in both manually signed languages and keyboard-token communications has indicated that non-repetitive, three-word sentences are a rarity (Terrace, 1979; Pinker, 1994). Dyadic associations? Yes. Triadic patterns? No. Both semantics and phonetics are not beyond the cognitive capacities of various species, but a cognitive barrier arises early in the realm of syntax, where the sequential ordering of *three* items plays an important role. Unlike human children (who rapidly progress from isolated words to two-, three-, and multi-word sentences), animals proceed to dyadic associations without an intrinsic sequential order – and their repetition. Failing to grasp the triadic principles of phrase structure – through which causality, as distinct from simple correlation, can be conveyed – grammatically “complex” linguistic structures remain a challenge to all species except *H. sapiens*.

## Tools

**FIGURE 7:**
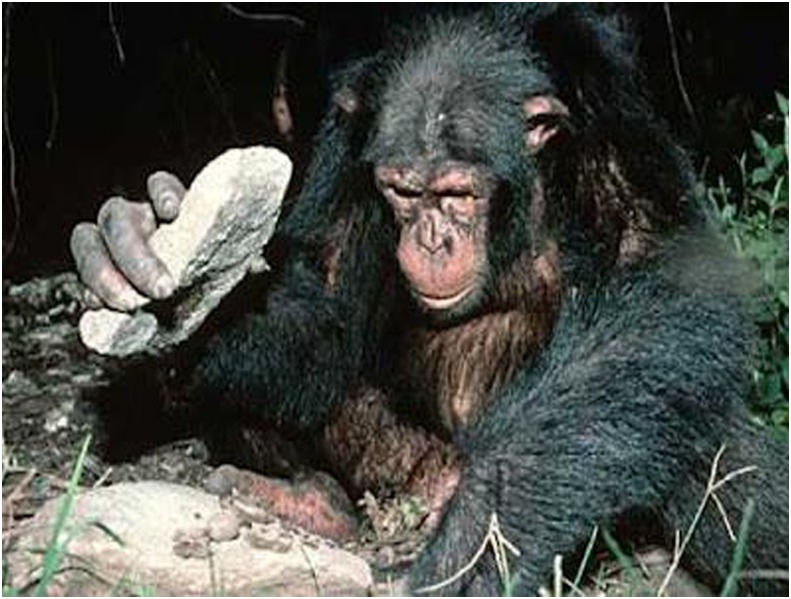
Chimpanzees have learned the trick of placing (i) a hard-shell nut on a (ii) suitably firm base and hammering it (iii) with a stone to get at the edible seed. The relationship among hammer, nut, and anvil is a triadic insight that is likely to have been mastered by our early ancestors more than 3 million years ago, but is a rarity in the animal kingdom. “There are several stages in learning how to crack nuts. First, learn how to handle one object. Then, try combining two objects. And finally, put all three together.” (screenshot from Uhlenbroek, 2008).

**FIGURE 8:**
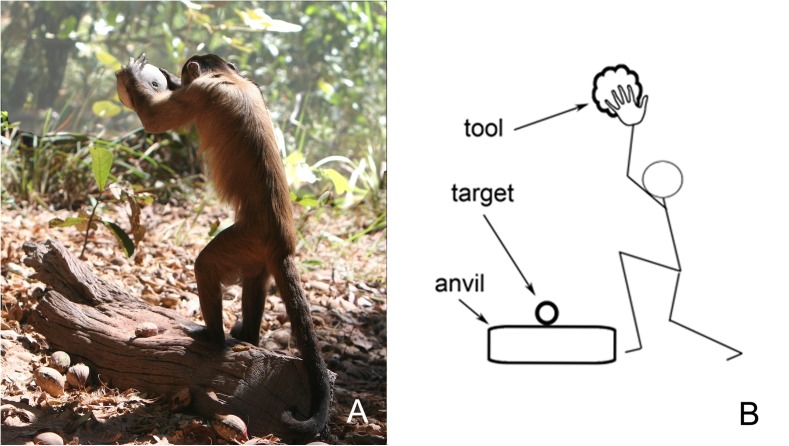
Capuchin monkeys in South America have also learned the inherently triadic skill of nut-cracking using hammer and anvil (screenshot from Jordan, 2009). **(A)** A capuchin monkey in action cracking open an edible nut. Both hands are required to handle the heavy stone. **(B)** The three items that the monkey must keep in mind for success.

The newest insights into cognitive triads have come from the oldest field of study concerned with human evolution: the construction and use of primitive stone-tools (Brandi et al., 2014). From observations on both chimpanzees (Carvalho et al., 2008) and capuchin monkeys (Boinski et al., 2008) in natural settings, it has been found that they too can use simple tools in cognitively complex ways. That is an ability that virtually nobody in the 20th century had thought possible. While skeptics might still question the cognitive sophistication of chimpanzees that use twigs to fish out termites from a nest or leaves to sponge up water, the use of a stone hammer to break open the shells of nuts placed on an anvil is an impressive skill with a triadic cognitive core (**Figures 7**, **8**).

Specifically, the “triadic perception” interpretation of rock-hammering (**Figure 8B**) is that these animals have learned skills in the wild that require consideration of not just one or two, but three objects simultaneously. One-object talents are not uncommon – and grabbing a morsel to eat is easy enough if the animal is equipped with a dexterous hand, beak or paw. Two-object talents, such as scooping out edible bugs with a stick, are far less common and require that the animal keep track of both the bugs and the stick. But three-object talents are extremely rare. Small groups of capuchin monkeys in South America, chimpanzees in Africa, and Pacific sea otters have spontaneously learned that placing (1) an edible nut with a hard shell onto (2) a firm, horizontal surface that will serve as an anvil, and striking it from above with (3) a hammer stone will result in a nutritious snack. Clearly, this task requires that the tool-user maintain *three* perceptually distinct objects in mind. Mastering the triadic skill demands practice over several years, and has been found to be transmitted from generation to generation (Boinski et al., 2008) – truly an unusual talent. While using an unmodified stone as a sledgehammer is of course still a very primitive example of tool usage, it appears that, by employing an anvil for successful execution, a small number of mammalian species have independently taken the first step into triadic cognition.

Calvin (1986) has emphasized the importance of throwing objects for human evolution. For both hunting and fishing with spears, the cognition underlying throwing is arguably an early evolutionary generalization of nut-cracking. The tool (rock or spear) and the target are similar to those in the capuchin skill, but the material “context” is the wind, air and distance that must be negotiated for determining the trajectory of the thrown object to reach the target.

## Social Cooperation

All of the topics discussed above are concerned with how information is processed within the brain of one individual, but most of the wonders of human civilization have been made possible by the cooperation of many individuals in pursuit of common goals. Say what one will about the relative intelligence of various species, the accomplishments of human cultures are beyond comparison with anything in the animal kingdom. And it is for this reason that many commentators on human evolution insist that the essence of our “specialness” is predominantly social (Saxe, 2006). Unlike most animals, we typically work together.

**FIGURE 9:**
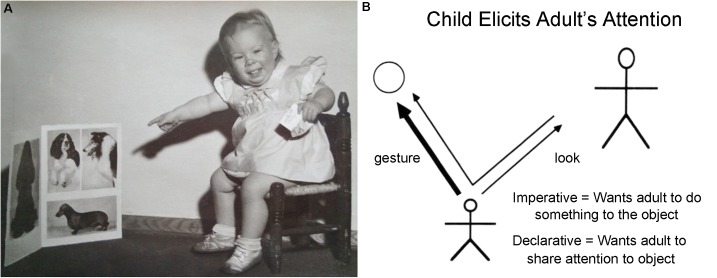
**(A)** Twenty-four month-old Sarah’s attempt at eliciting “joint attention” on a topic that is distinct from either parent or child. **(B)** A depiction of the triadic interaction between child, adult, and object by Tomasello et al. (2005). “Such cognition requires representing triadic relations: *You* and *Me*, collaboratively looking at, working on, or talking about *This*.” (Saxe, 2006).

If indeed we are not only tool-users and language-users (and artists and musicians), but even more importantly social beings, just what is the cognitive trick that has allowed us to master the art of social cooperation? The key is not simply empathetic recognition of our own species (as indeed all animal species are instinctually capable of), but rather the employment of, once again, fundamentally triadic cognitive mechanisms. Human beings normally and habitually empathize with other human beings by “reading their minds,” speculating on the other’s motivations, and then acting accordingly (Tomasello, 1999, 2003; Gomez, 2004; Saxe et al., 2004; Tomasello et al., 2005; Saxe, 2006; Baron-Cohen et al., 2013). The elements of these social interactions have been studied primarily in developmental psychology under the label of “joint attention,” where it is found that, from an early age, human infants follow the parental gaze in search of a topic for interaction. Preverbal infants soon use gaze and finger pointing in order to draw the attention of parents toward topics of mutual interest, and gradually come to understand joint activity, sharing and taking turns (**Figure 9**). Although it is clearly a long journey to the building of empires, the products of human civilization are, without exception, consequences of large-scale, prolonged social cooperation. As others have coherently demonstrated, social cooperation is, for each participating individual, cognitively triadic.

## Conclusion

I have outlined the hypothesis that the core talents underlying the “higher” cognition in diverse fields of human psychology are a consequence of three-way mental associations. Such cognition is rare among animals, in general (although not entirely unprecedented, especially among Primates), but is the essence of the way in which human beings normally and habitually “think.” A simpler form of cognition, i.e., one-to-one associations, is essentially dyadic. Dyadic cognition is of course extremely useful and an understanding of correlations is often sufficient for the purposes of biological survival (avoid the toxic smell, approach the friendly smile, etc.). In contrast, triadic processes require an additional cognitive effort – not merely a third element, but a tripling of the dyadic associations inherent to a triad of cues and the introduction of an “emergent property” in the form of a novel three-way pattern. That complexity produces an unprecedented and notably slow “mindfulness” that is the unspoken pride and joy – and arguably the source of much disputation – for humankind. Unlike the two-element associational processes, of which all animal nervous systems are capable, a triad involves an association between, to begin with, two elements, but now with a minimal context provided by the third element. The dyadic association then becomes context-dependent, and the meaning of the dyadic relationship is necessarily affected by the context. As a consequence, by their very nature, triads are *not* simply the summation of several dyads, but are themselves primitives with their own meaning that can then be employed in more complex associational phenomena.

In that respect, it is relevant to note that cognitive dyads are additive, but triads are inherently recursive and hierarchical, insofar as each element in a triad can itself be replaced by a triad – leading to conceptual structures the complexity of which is limited only by the capacity of short-term memory. The recursive nature of language is well known and easily studied using imbedded relative clauses. Dunbar (2007), for example, argues that normal human beings have the capacity for five-level syntactic recursion (intentionality), beyond which the burden on short-term memory impairs performance. Similarly-triadic recursion is a known feature of music, where the triadic tricks of harmonic mode are repeatedly utilized to construct harmonic cadences that produce a well-defined affective mood. In pictorial art, the overall coherency of a realistic visual scene relies heavily on employing shading, shadows, and perspective cues that are consistent with a unique vantage point from which the visual scene is depicted. And, most well known of all, the recursive imbedding of gadgets and gizmos in the construction of tools has increased since the Paleolithic Era and accelerated dramatically since the industrial revolution. Knives and hammers are tools whose construction and use are easily understood in a triadic framework, but the vast majority of modern tools can be understood only in the context of multiple-imbedding. The recursive implementation of cognitive triads then leads to the amazing complexity of “real” language, “real” music, “real” art, “real” tools, and “real” social interactions – but the cognitive core is simply triadic relationships.

The hypothesis of triadic cognition still requires considerable refinement. The five categories discussed above are the best known, most-widely discussed candidates of “higher” human cognition, but there may be other multimodal or unimodal triadic operations involving, for example, purely proprioceptive (athletics) or gustatory (cuisine) cues. More precise definitions of the relationships among cues (and the cognitive chunking of cues into smaller sets with fewer relationships) will also be needed. Finally, reformulation of the familiar concepts of “folk” psychology in terms of the formal “ternary operations” of Boolean algebra will undoubtedly be necessary. Already we know that any ternary operation can be stated in terms of the logistician’s definitions of “meet,” “join,” and “complement” (e.g., Givant and Halmos, 2009). In principle, combinations of those operations provide the entire logical framework for a rigorous neuroscience of triadic cognition, but the Boolean algebra in cognition will likely prove to be the easy half of the task. The hard half will be the reduction of currently, poorly defined psychological phenomena to their cognitive essences.

Normal human beings can easily juggle three quasi-independent stimuli (visual cues, tones, words, objects, or mental perspectives) in exercising the talents discussed above. By taking the relationships among all three stimuli into consideration simultaneously, we find meaning in the three-way interaction that is not apparent from the summation of multiple two-way interactions. In contrast, while animals can accurately perceive the same sensory stimuli and learn the same dyadic associations between pairs of stimuli, they apparently find no inherent meaning in the triad itself. Because of the stark difference between dyadic associations and triadic relations, it appears that triadic processing – breaking the world into bite-size triads – is the essence of human *intelligence*. It is this trick that has allowed our species to sometimes transcend the dictates of strictly dyadic, correlational behaviorism and to enjoy the mindfulness of higher-order, context-dependent, cognitive complexity.

## Author Contributions

The author confirms being the sole contributor of this work and approved it for publication.

## Conflict of Interest Statement

The author declares that the research was conducted in the absence of any commercial or financial relationships that could be construed as a potential conflict of interest.

## Figures and Tables

**FIGURE A1:**
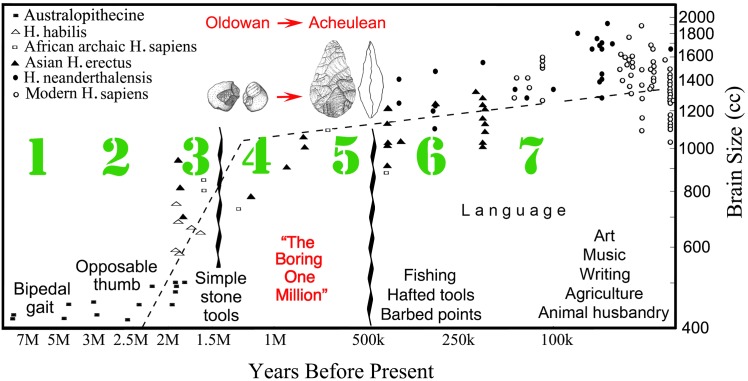
At least six subspecies of our early ancestors from Africa and the Middle East are well represented in the fossil record. Collectively, they show a remarkable increase in brain size from the chimpanzee (400 cc) to modern *Homo sapiens* (1400 cc) – over a period of 7 million years. During that period, no other African mammal showed *comparable increases in brain size*! Particularly difficult for paleontologists to explain is the era known as “the boring one million years.” This was when human brain expansion was most vigorous and yet the changes in stone-tool structure seen in the transition from the Oldowan toolkit to the Acheulean toolkit were extremely modest. Complex hafted tools (consisting of two or more components) are not found until much later, but one noteworthy change in the emergence of Acheulean tools was the establishment of cerebral dominance. That development is inferred from microscopic analysis of the ballistic strikes needed to produce tools, and indicate that our ancestors who crafted the Acheulean hand-axes were right-handed (McManus, 2002). Insofar as such tool-making entails prolonged motor training for the appropriate removal of flakes from the core, it is inconceivable that hand-ax makers would have alternated between left and right hands. Training of one hand (and the motor and premotor cortex of the contralateral cerebral hemisphere) would have been a sufficient challenge without the additional chore of achieving ambidexterity. In this regard, “the boring one million years” is likely to have been a period of consolidation of the dominance of the left cerebral hemisphere – i.e., the organization of executive motor functions in *one* hemisphere, while freeing the other hemisphere to specialize in other tasks (Figure adapted from Oppenheimer, 2003, p. 17).

## Footnotes

1 A century and a half of research following Darwin’s theory of evolution has revealed many similarities in cognition among diverse animal species, but there remains a huge quantitative “gap” (Suddendorf, 2013) in capabilities that I believe is suggestive of a qualitatively distinct mode of information-processing in the human brain. Few researchers in the neurosciences would defend the idea of a non-neuronal component in animal or human cognition, but the empirical reality of the cognitive “gap" demands a coherent, materialist explanation that has not previously been advocated.

2 The difficulty of defending a thesis concerning the importance of triadic vs. dyadic processes is that arguments need to be developed in the most controversial fields concerned with human behavior – where debate is most intense, empirical research is most abundant, and, correspondingly, theoretical stances are most staunchly defended. In the present essay, my intention is merely to point to the well-established triadic arguments made by specialists in their respective fields. Defending the overall triadic argument as a possible explanation of human cognition is the theoretical challenge I undertake, but the implicitly or explicitly triadic arguments of many others provide the foundation for the generalized argument. In that respect, the present hypothesis is inherently controversial, but it is built upon the strong foundations of the various “triadic models” advocated by others over the course of many decades.

## Appendix

### Appendix: The Timeline of Human Evolution

A plausible scenario for the sequence of events that led to the cognition of modern *H. sapiens* is shown in **Appendix A1** and can be summarized as “The Seven Steps to Modernity.” While precise dates are not known and many details are missing, these seven stages are fully consistent with the chronology of the empirical fossil record.

#### The Seven Steps to Modernity:

##### (Step 0) Climate change

During a series of ice ages that struck northern Europe some 7–8 million years ago, our Primate ancestors in central/east Africa experienced arid conditions that transformed bountiful jungles into less bountiful woodlands and savannahs. The paucity of fruit-bearing trees made their normal arboreal existence and vegetarian diet impossible, and led to:

##### (Step 1) Bipedal Locomotion

Being inherently slower than quadrupedal locomotion, the bipedal hominid, *Australopithecine*, found itself at a disadvantage in relation to predatory carnivores (Tattersall, 2002, p. 15). Despite being somewhat slower, those hunter/gatherer ancestors thrived, probably as a consequence of the unprecedented advantages of:

##### (Step 2) Dexterous Hands

In contrast to the radical changes in the pelvis and spine that were required for bipedalism, the fossil record shows only small changes in the morphology of the hands of *Homo habilis*, as the hands themselves were employed, in effect, as tools (Oppenheimer, 2003). Limbs that had previously been used primarily for jungle agility could now be employed for new purposes: carrying and manipulating objects. The dexterity of hands with powerful opposable thumbs was eventually exploited in the invention of:

##### (Step 3) Simple Stone Tools

The earliest tools of the so-called Oldowan type exhibit little more than a sharp edge, but that was enough for the purposes of scavenging the meat and hides of megafauna (Stringer, 2012). Following upon the behavioral diversity implied by simple tool usage, the improved nutrition provided by meat-eating allowed for huge increases in brain volume (Wrangham, 2009; Herculano-Houzel, 2016). Having many more neurons in the central nervous system was certainly beneficial in allowing for greater cognitive complexity, but the true significance lay in:

##### (Step 4) The Expansion of the Neocortex

This period of brain enlargement occurred at a time when there were few changes in the morphology of tools – a period nicknamed by paleoanthropologists as “the boring one million years.” The prolonged era of behavioral stagnancy was first noted by Jelinek (1977), but has since been endorsed by Tattersall (2002, 2012), p. 104, p. 42, Coolidge and Wynn (2009), pp. 155–156, Stringer (2012), p. 244, and Suddendorf (2013), p. 253. The enigma is that it is hard to understand how new tools would *not* be developed (given the already-established basic stone tool technology of hammer and core), and yet there were essentially no technological innovations during this millennium of millennia. Oppenheimer (2003), p. 23 has argued that the most significant mutation event ever to occur in the evolution of *Homo sapiens* took place at the advent of the Oldowan era – a developmental change that produced brain enlargement, in general, and expansion of the cerebral neocortex, in particular. While paleoanthropologists have noted that surprisingly few new behaviors accompanied the increase in brain volume, there was nonetheless a remarkable change in brain morphology that has since influenced all subsequent human evolution (Zaidel and Iacoboni, 2003). That is, the transition from Oldowan to Acheulean tools was accompanied by the emergence of:

##### (Step 5) Lateralized Cerebral Dominance and Handedness

Note that an Oldowan tool can be created with a mere 1 ∼ 6 ballistic strikes to a core stone, whereas the Acheulean hand-ax cannot be produced with less than 50 strikes (and probably many more) of similar strength, force, and orientation at appropriate sites on the core. The *qualitative* conclusion drawn from such a simple *quantitative* finding is that the makers of Acheulean tools were necessarily “handed” – not ambidextrous, because of the need to train one hand. The creation of hand-axes by alternating between the left and right hands would have demanded twice the time to train the motor cortex of both hemispheres, whereas the consistent use of one hand would have been more efficient – both today and 2 million years ago. The “boring one million years” may therefore have been “boring” from a behavioral perspective, but nevertheless a time during which the specialization of one cerebral hemisphere for motor dominance in specifically tool creation was consolidated (Frost, 1980). Alone, the species-level preference for using the right hand when striking a core stone to produce sharp-edged flakes might have had little significance for human evolution, but the prolonged era of the motor dominance of the right hand (left hemisphere) was followed by:

##### (Step 6) Lateralized Cerebral Specialization

Unilateral motor dominance was important for the training of the favored hand in the motor skills needed for producing stone tools, but particularly noteworthy was the liberation of the *contralateral* motor cortex from the training of motor skills. That freedom made possible the specialization of the frontal neocortex of the right hemisphere for other forms of cognition (Jaynes, 1976; Cook, 1986; Hugdahl and Westerhausen, 2010). Early “non-dominant” cerebral hemisphere talents would have included understanding the visuospatial geometrical constraints of creating an Acheulean hand-ax and maintaining a visual image of the intended product “in mind” – talents reminiscent of modern-day right hemisphere skills. Having thus developed a dual-control neuronal mechanism for the construction of tools, *Homo sapiens* with functionally lateralized brains subsequently adapted the dual control architecture in the supreme motor behavior of our species:

##### (Step 7) Spoken Language

In the modern human brain, the single most unambiguous aspect of functional brain asymmetry is that found for speech. Although mixed dominance is often found for language perception, semantics, and prosody, the need for unilateral executive control over motor output (speech) remains uncompromising: fully 97% of right-handers and 80% of left-handers exhibit unilateral motor control over the organs of speech (Warrington and Pratt, 1973). Conversely, the absence of unilateral functional dominance during speech is associated with stuttering (e.g., Watkins et al., 2008). The significance of hemispheric dominance and lateralized specialization for virtually every other aspect of human psychology remains controversial (Hugdahl and Westerhausen, 2010; Ocklenburg et al., 2016), but the asymmetrical activation of the cerebral hemispheres during language production is the rule rather than the exception in *Homo sapiens*. There are few indications in the fossil record concerning precisely when language emerged, but it is thought unlikely to have predated the making of simple hafted tools. What that implies is that art, science, and technology have blossomed worldwide in the remarkably short period of two or three thousand years following the emergence of the precise sequentialization of unilateral motor commands for both speech and tool-making.

These seven steps leading to modern cognition can be succinctly stated as follows: Step 1 freed the hands from the chores of locomotion. Step 2 was the emergence of dexterous hands capable of manipulating the available raw materials of stone, wood, animal hides, and plant fiber. Step 3 was the nutritional gain that primitive tools made possible through meat-eating. Step 4 was the subsequent brain enlargement, producing relatively large regions of polymodal association cortex. Step 5 was the beginning of the heavily repetitive manual activity of stone tool manufacture that required the training of a dominant hand (cerebral hemisphere) for executive motor functions. Step 6 was the emergence of non-dominant (right) hemisphere specializations that were unrelated to motor skills, but were relevant to the cognitive processing of affective and visuospatial information. And Step 7 was the development of the dual cognitive functions of spoken language in the left hemisphere and contextual processing in the right hemisphere (Geschwind, 1965). It is this combination of executive skills together with paralinguistic, affective and contextual functions that are today considered to be the essence of human “intelligence.”

## References

[B1] Addis L. (1999). *Of Mind and Music.* Ithaca, NY: Cornell University Press.

[B2] Arnheim R. (1974). *Art and Visual Perception: A Psychology of the Creative Eye.* Berkeley, CA: University of California Press.

[B3] Ball P. (2010). *The Music Instinct.* London: The Bodley Head.

[B4] Baron-Cohen S. Tager-Flusberg H. Lombardo M. V. (eds) (2013). *Understanding Other Minds: Perspectives from Developmental Social Neuroscience.* Oxford: Oxford University Press 10.1093/acprof:oso/9780199692972.001.0001

[B5] Baxandall M. (1995). *Shadows and Enlightenment.* New Haven, CT: Yale University Press.

[B6] Berwick R. C. Chomsky N. (2016). *Why Only Us.* Cambridge, MA: MIT Press 10.7551/mitpress/9780262034241.001.0001

[B7] Bickerton D. (1990). *Language and Species.* Chicago, IL: University of Chicago Press.

[B8] Bickerton D. (2014). *More Than Nature Needs: Language, Mind, and Evolution.* New York, NY: Harvard University Press 10.4159/9780674728523

[B9] Blacking J. (1973). *How Musical Is Man?* Seattle, WA: University of Washington Press.

[B10] Boeckx C. (2006). *Linguistic Minimalism: Origins, Concepts, Methods, and Aims.* Oxford: Oxford University Press.

[B11] Boinski S. Quatrone R. P. Swartz H. (2008). Substrate and tool use by brown capuchins in Suriname: ecological contexts and cognitive bases. *Am. Anthropol.* 102 741–761. 10.1525/aa.2000.102.4.741

[B12] Brandi M. L. Wohlschläger A. Sorg C. Hermsdörfer J. (2014). The neural correlates of planning and executing actual tool use. *J. Neurosci.* 34 13183–13194. 10.1523/JNEUROSCI.0597-14.2014 25253863 PMC6608341

[B13] Brown D. E. (1991). *Human Universals.* New York, NY: McGraw-Hill.

[B14] Calvert G. Spence C. Stein B. E. (eds) (2004). *The Handbook of Multisensory Processes.* Cambridge, MA: MIT Press.

[B15] Calvin W. H. (1986). *The River that Flows Uphill.* New York, NY: Macmillan.

[B16] Carnie A. Guilfoyle E. (eds) (2000). *The Syntax of Verb Initial Languages.* Oxford: Oxford University Press.

[B17] Carvalho S. Cunha E. Sousa C. Matsuzawa T. (2008). Chaînes opératoires and resource-exploitation strategies in chimpanzee (*Pan troglodytes*) nut cracking. *J. Hum. Evol.* 55 148–163. 10.1016/j.jhevol.2008.02.005 18359504

[B18] Casati R. (2004). *Shadows: Unlocking their Secrets from Plato to our Time.* New York, NY: Vintage Books.

[B19] Chatterjee A. (2014). *The Aesthetic Brain: How we Evolved to Desire Beauty and Enjoy Art.* New York, NY: Oxford University Press.

[B20] Chomsky N. (1965). *Aspects of the theory of Syntax.* Cambridge, MA: MIT Press.

[B21] Chomsky N. (2000). *New Horizons in the Study of Language and Mind.* New York, NY: Cambridge University Press 10.1017/CBO9780511811937

[B22] Cook N. D. (1986). *The Brain Code: Mechanisms of Information Transfer and the Role of the Corpus Callosum.* London: Methuen Publishing.

[B23] Cook N. D. (2002). *Tone of Voice and Mind.* Amsterdam: John Benjamins Publishing Company 10.1075/aicr.47

[B24] Cook N. D. (2009). Harmony perception: harmoniousness is more than the sum of interval consonance. *Music Percept.* 27 25–41. 10.1525/mp.2009.27.1.25

[B25] Cook N. D. (2012). *Harmony, Perspective and Triadic Cognition.* New York, NY: Cambridge University Press.

[B26] Cook N. D. (2017). Calculation of the acoustical properties of triadic harmonies. *J. Acoust. Soc. Am.* 142 3748–3755. 10.1121/1.5018342 29289060

[B27] Cook N. D. Fujisawa T. X. (2006). The psychophysics of harmony perception: harmony is a 3-tone phenomenon. *Empir. Musicol. Rev.* 1 106–131. 10.18061/1811/24080

[B28] Cook N. D. Fujisawa T. X. Takami K. (2006). Evaluation of the affective valence of speech using pitch substructure. *IEEE Trans. Audio Speech Lang. Process.* 14 142–155. 10.1109/TSA.2005.854115

[B29] Cook N. D. Hayashi T. (2008). The psychoacoustics of musical harmony. *Am. Sci.* 96 311–319. 10.1511/2008.73.3845

[B30] Cook N. D. Hayashi T. Amemiya T. Suzuki K. Leumann L. (2002). The effects of visual field inversions on the reverse perspective illusion. *Perception* 31 1147–1151. 10.1068/p3336 12375877

[B31] Cook N. D. Yutsudo A. Fujimoto N. Murata M. (2008a). Factors contributing to depth perception. *Spat. Vis.* 21 397–411. 10.1163/156856808784532518 18534111

[B32] Cook N. D. Yutsudo A. Fujimoto N. Murata M. (2008b). On the importance of three visual cues for perceiving 3D depth in 2D images. *Emp. Stud. Arts* 26 67–83.

[B33] Coolidge F. L. Wynn T. (2009). *The Rise of Homo Sapiens: The Evolution of Modern Thinking.* New York, NY: Wiley-Blackwell 10.1002/9781444308297

[B34] Cooper G. Meyer L. B. (1960). *The Rhythmic Structure of Music.* Chicago, IL: Chicago of University Press.

[B35] Corballis M. C. (2011). *The Recursive Mind: The Origins of Human Language, thought, and Civilization.* Princeton, NJ: Princeton University Press.

[B36] Cowan N. (2001). The magical number 4 in short-term memory: a reconsideration of mental storage capacity. *Behav. Brain Sci.* 24 87–114. 10.1017/S0140525X01003922 11515286

[B37] Curtis G. (2006). *The Cave Painters: Probing the Mysteries of the World’s First Artists.* New York, NY: Anchor.

[B38] Damisch H. (1993). *The Origin of Perspective.* Cambridge, MA: MIT Press.

[B39] Davies S. (2012). *The Artful Species: Aesthetics, Art, and Evolution.* Oxford: Oxford University Press 10.1093/acprof:oso/9780199658541.001.0001

[B40] Deacon T. W. (1997). *The Symbolic Species.* New York, NY: W. W. Norton.

[B41] Desain P. Honing H. (2003). The formation of rhythmic categories and metric priming. *Perception* 32 341–365. 10.1068/p3370 12729384

[B42] Donald M. (2001). *A Mind So Rare: The Evolution of Human Consciousness.* New York, NY: W. W. Norton.

[B43] Dunbar R. (2007). “Why are human not just great apes?,” in *What Makes us Human?*, ed. Pasternak C. (Oxford: One World Publications), 37–48.

[B44] Dunbar R. (2016). *Human Evolution: Our Brains and Behavior.* New York, NY: Oxford University Press.

[B45] Dutton D. (2009). *The Art Instinct: Beauty, Pleasure and Human Evolution.* London: Bloomsbury Publishing.

[B46] Edgertown S. Y. (2009). *The Mirror, the Window, and the Telescope: How Renaissance Linear Perspective Changed our Vision of the Universe.* Ithaca, NY: Cornell University Press.

[B47] Evans V. (2014). *The Language Myth: Why Language is not an Instinct.* New York, NY: Cambridge University Press 10.1017/CBO9781107358300

[B48] Frost G. T. (1980). Tool behavior and the origins of laterality. *J. Hum. Evol.* 9 447–459. 10.1016/0047-2484(80)90002-0

[B49] Fujisawa T. X. Cook N. D. (2011). The perception of harmonic triads: an fMRI study. *Brain Imaging Behav.* 5 109–125. 10.1007/s11682-011-9116-5 21298563

[B50] Geschwind N. (1965). Dysconnexion syndromes in animals and man. *Brain* 88 237–294. 10.1093/brain/88.2.237 5318481

[B51] Givant S. Halmos P. (2009). *Introduction to Boolean Algebras.* New York, NY: Springer.

[B52] Glasser M. F. Coalson T. S. Robinson E. C. Hacker C. D. Harwell J. Yacoub E. (2016). A multi-modal parcellation of human cerebral cortex. *Nature* 536 171–181. 10.1038/nature18933 27437579 PMC4990127

[B53] Gombrich E. H. (1961). *Art and Illusion: A Study in the Psychology of Pictorial Representation.* Princeton, NJ: Princeton University Press.

[B54] Gombrich E. H. (1995). *Shadows: The Depiction of Cast Shadows in Western Art.* New Haven, CT: Yale University Press.

[B55] Gomez J. C. (2004). *Apes, Monkeys, Children, and the Growth of Mind.* New York, NY: Harvard University Press.

[B56] Gweon H. Saxe R. (2013). “Developmental neuroscience theory of mind,” in *Neural Circuit Development and Function in the Brain: Comprehensive Developmental Neuroscience* Vol. 3 eds Rubenstein J. Rakic P. (New York, NY: Elsevier), 367–377. 10.1016/B978-0-12-397267-5.00057-1

[B57] Hayashi T. Umeda C. Cook N. D. (2007). An fMRI study on the reverse perspective illusion. *Brain Res.* 1163 72–78. 10.1016/j.brainres.2007.05.073 17631871

[B58] Hecht H. Schwartz R. Atherton M. (eds) (2003). *Looking into Pictures: An Interdisciplinary Approach to Pictorial Space.* Cambridge, MA: MIT Press.

[B59] Heilbron J. L. (2010). *Galileo.* Oxford: Oxford University Press.

[B60] Herculano-Houzel S. (2016). *The Human Advantage: How our Brains Became Remarkable.* Cambridge, MA: MIT Press 10.7551/mitpress/9780262034258.001.0001

[B61] Hoeschele M. Cook R. G. Guillette L. M. Brooks D. I. Sturdy C. B. (2012). Black-capped chickadee (*Poecile atricapillus*) and human (*Homo sapiens*) chord discrimination. *J. Comp. Psychol.* 126 57–67. 10.1037/a0024627 21942569

[B62] Hugdahl K. Westerhausen R. (eds) (2010). *The Two Halves of the Brain: Information Processing in the Cerebral Hemispheres.* Cambridge, MA: MIT Press 10.7551/mitpress/9780262014137.001.0001

[B63] Huron D. (2006). *Sweet Anticipation: Music and the Psychology of Expectation.* Cambridge, MA: MIT Press.

[B64] Jackendoff R. (2002). *Foundations of Language: Brain, Meaning, Grammar, Evolution.* Oxford: Oxford University Press 10.1093/acprof:oso/9780198270126.001.0001 15377127

[B65] Jaynes J. (1976). *The Origins of Consciousness in the Breakdown of the Bicameral Mind.* Boston, MA: Houghton Mifflin.

[B66] Jelinek A. J. (1977). The lower paleolithic: current evidence and interpretations. *Annu. Rev. Anthropol.* 6 21–44. 10.1146/annurev.an.06.100177.000303

[B67] Johnson-Frey S. H. (2004). The neural bases of complex tool use in humans. *Trends Cogn. Sci.* 8 71–78. 10.1016/j.tics.2003.12.002 15588811

[B68] Jonides J. Lewis R. L. Nee D. E. Lustig C. A. Berman M. G. Moore K. S. (2008). The mind and brain of short-term memory. *Ann. Rev. Psychol.* 59 193–224. 10.1146/annurev.psych.59.103006.093615 17854286 PMC3971378

[B69] Jordan T. (2009). *Capuchin Monkey Nut Cracking Tool Use.* Available at: https://www.youtube.com/watch?v=_MgHBvp1uwk [accessed 7 June, 2017].

[B70] Kastner M. P. Crowder R. G. (1990). Perception of major/minor: emotional connotations in young children. *Music Percept.* 8 189–202. 10.2307/40285496

[B71] Kemp M. (1990). *The Science of Art: Optical themes in Western Art from Brunelleschi to Seurat.* New Haven, CT: Yale University Press.

[B72] Klein R. G. Edgar B. (2002). *The Dawn of Human Culture.* New York, NY: Wiley.

[B73] Kubovy M. (1986). *The Psychology of Perspective and Renaissance Art.* New York, NY: Cambridge University Press 10.1086/509092

[B74] Lieberman P. (2007). The evolution of human speech. *Curr. Antrhopol.* 48 39–66.

[B75] Massey L. (2003). *The Treatise on Perspective: Published and unpublished.* New Haven, CT: Yale University Press.

[B76] McManus C. (2002). *Right Hand, Left Hand.* New York, NY: Harvard University Press.

[B77] Meyer L. B. (1956). *Emotion and Meaning in Music.* Chicago, IL: Chicago University Press.

[B78] Mithen S. J. (1996). *The Prehistory of the Mind: The Cognitive Origins of Art, Religion and Science.* London: Thames and Hudson.

[B79] Mithen S. J. (2005). *The Singing Neanderthals: the Origins of Music, Language, Mind and Body.* London: Weidenfeld & Nicolson.

[B80] Murray M. M. Wallace M. T. (eds) (2012). *The Neural Bases of Multisensory Processes.* Roca Baton, FL: CRC Press. 22593873

[B81] Narmour E. (1990). *The Analysis and Cognition of Basic Melodic Structures.* Chicago, IL: University of Chicago Press.

[B82] Oakes L. M. Bauer P. J. (2007). *Short- and Long-Term Memory in Infancy and Early Childhood.* New York, NY: Oxford University Press. 10.1515/revneuro-2015-0052

[B83] Ocklenburg S. Friedrich P. Gunturkun O. Genc E. (2016). Intrahemispheric white matter asymmetries: the missing link between brain structure and functional lateralization? *Rev. Neurosci.* 27 111–123. 10.1515/revneuro-2015-0052 26812865

[B84] Oppenheimer S. (2003). *Out of Eden: The Peopling of the World.* New York, NY: Robinson & Yablon, P.C.

[B85] Panofsky E. (1997). *Perspective as Symbolic Form.* London: Zone Books 10.1007/978-3-642-74831-8

[B86] Parncutt R. (1989). *Harmony: A psychoacoustical Approach.* Berlin: Springer.

[B87] Pasternak C. (ed.) (2007). *What Makes us Human?* Oxford: Oneworld Publications.

[B88] Pepperberg I. M. (1999). *The Alex Studies: Cognitive and Communicative Abilities of Grey Parrots.* New York, NY: Harvard University Press.

[B89] Pinker S. (1994). *The Language Instinct: How the Mind Creates Language.* New York, NY: Morrow Inc, 339.

[B90] Plaisier M. A. Kappers A. M. L. (2016). Numerosity perception in the various sensory modalities. *Perception* 45 67–78. 10.1121/1.1909741

[B91] Plomp R. Levelt W. J. M. (1965). Tonal consonances and critical bandwidth. *J. Acoust. Soc. Am.* 38 548–560. 5831012 10.1121/1.1909741

[B92] Pollard C. Sag I. (1994). *Head-Driven Phrase Structure Grammar.* Chicago, IL: University of Chicago Press.

[B93] Purves D. Lotto B. (2003). *Why we see What we do: An Empirical theory of Vision.* Sunderland, MA: Sinauer Associates.

[B94] Roberts L. (1986). Consonant judgments of musical chords by musicians and untrained listeners. *Acustica* 62 163–171.

[B95] Savage-Rumbaugh E. S. Shanker S. G. Taylor T. J. (2001). *Apes, Language, and the Human Mind.* Oxford: Oxford University Press. 10.1016/j.conb.2006.03.001

[B96] Saxe R. (2006). Uniquely human social cognition. *Curr. Opin. Neurobiol.* 16 235–239. 10.1146/annurev.psych.55.090902.142044 16546372

[B97] Saxe R. Carey S. Kanwisher N. (2004). Understanding other minds: linking developmental psychology and functional neuroimaging. *Ann. Rev. Psychol.* 55 87–124. 14744211 10.1146/annurev.psych.55.090902.142044

[B98] Schoenberg A. (1978). *Theory of Harmony.* Berkeley, CA: University of California Press.

[B99] Sethares W. A. (2005). *Tuning, Timbre, Spectrum, Scale.* Berlin: Springer.

[B100] Solso R. L. (2003). *The Psychology of Art and the Evolution of the Conscious Brain.* Cambridge, MA: MIT Press.

[B101] Stoichita V. I. (1997). *A Short History of the Shadow.* London: Reaktion Books.

[B102] Storr A. (1992). *Music and the Mind.* New York, NY: Ballantine Books.

[B103] Stringer C. (2012). *The Origin of our Species.* New York, NY: Penguin Group, 244.

[B104] Suddendorf T. (2013). *The Gap: The Science of what Separates us from other Animals.* New York, NY: Basic Books.

[B105] Tattersall I. (1998). *Becoming Human: Evolution and Human Uniqueness.* New York, NY: Harcourt Brace.

[B106] Tattersall I. (2002). *The Monkey in the Mirror: Essays on the Science of what makes us Human.* New York, NY: Harcourt.

[B107] Tattersall I. (2007). “Human evolution and the human condition,” in *What Makes us Human?*, ed. Pasternak C. (Oxford: Oneworld Publications), 133–145.

[B108] Tattersall I. (2012). *Masters of the Planet: The Search for our Human Origins.* New York, NY: Macmillan Publishing.

[B109] Temperley D. (2007). *Music and Probability.* Cambridge, MA: MIT Press 10.1901/jeab.1979.31-161

[B110] Terrace H. S. (1979). Is problem solving language? *J. Exp. Anal. Behav.* 31 161–175.

[B111] Tomasello M. (1999). *The Cultural Origins of Human Cognition.* New York, NY: Harvard University Press.

[B112] Tomasello M. (2003). *Constructing a Language: A Usage-Based theory of Language Acquisition.* New York, NY: Harvard University Press 10.4159/9780674726369

[B113] Tomasello M. (2014). *A Natural History of Human Thinking.* New York, NY: Harvard University Press. 10.1017/S0140525X05000129

[B114] Tomasello M. Carpenter M. Call J. Behne T. Moll H. (2005). Understanding and sharing intentions: the origins of cultural cognition. *Behav. Brain Sci.* 28 675–691. 10.1093/acprof:oso/9780195306361.001.0001 16262930

[B115] Turner M. (ed.) (2006). *The Artful Mind: Cognitive Science and the Riddle of Human Creativity.* Oxford: Oxford University Press.

[B116] Tymoczko D. (2011). *A Geometry of Music: Harmony and Counterpoint in the Extended Common Practice.* Oxford: Oxford University Press.

[B117] Uhlenbroek C. (2008). *Chimpanzees’ Sophisticated use of Tools – BBC Wildlife.* Available at: https://www.youtube.com/watch?v=5Cp7_In7f88 [assessed 7 June, 2017].

[B118] von Helmholtz H. (1885/1954). *On the Sensations of Tone.* New York, NY: Longmans, Green 10.1016/0028-3932(73)90029-8

[B119] Warrington E. K. Pratt R. T. (1973). Language laterality in left-handers assessed by unilateral E.C.T. *Neuropsychologia* 11 423–428. 10.1093/brain/awm241 4758185

[B120] Watkins K. E. Smith S. M. Davis S. Howell P. (2008). Structural and functional abnormalities of the motor system in developmental stuttering. *Brain* 131 50–59. 17928317 10.1093/brain/awm241 PMC2492392

[B121] Whiten A. (1999). “The evolution of deep social mind in humans,” in *The Descent of Mind*, Corballis M. C. Lea S. E. G. (Oxford: Oxford University Press).

[B122] Wolfe T. (1975). *The Painted Word.* New York, NY: Farrar, Straus & Giroux.

[B123] Wrangham R. (2009). *Catching Fire: How Cooking made us Human.* New York, NY: Basic Books.

[B124] Zaidel E. Iacoboni M. (eds) (2003). *The Parallel Brain: The Cognitive Neuroscience of the Corpus Callosum.* Cambridge, MA: MIT Press.

[B125] Zeki S. (1999). *Inner Vision: An Exploration of Art and the Brain.* Oxford: Oxford University Press.

